# Genetic Variants and Their Associations to Type 2 Diabetes Mellitus Complications in the United Arab Emirates

**DOI:** 10.3389/fendo.2021.751885

**Published:** 2022-01-06

**Authors:** Sarah ElHajj Chehadeh, Noura S. Sayed, Hanin S. Abdelsamad, Wael Almahmeed, Ahsan H. Khandoker, Herbert F. Jelinek, Habiba S. Alsafar

**Affiliations:** ^1^ Khalifa University Center of Biotechnology, Abu Dhabi, United Arab Emirates; ^2^ Biomedical Engineering Department, Khalifa University of Science and Technology, Abu Dhabi, United Arab Emirates; ^3^ College of Medicine and Health Sciences, Khalifa University of Science and Technology, Abu Dhabi, United Arab Emirates; ^4^ Institute of Cardiac Science, Sheikh Khalifa Medical City, Abu Dhabi, United Arab Emirates; ^5^ Heart and Vascular Institute, Cleveland Clinic, Abu Dhabi, United Arab Emirates; ^6^ Healthcare Engineering Innovation Center (HEIC), Khalifa University of Science and Technology, Abu Dhabi, United Arab Emirates

**Keywords:** type 2 diabetes mellitus, retinopathy, nephropathy, peripheral neuropathy, microvascular complication, single nucleotide polymorphism, United Arab Emirates, Arab population

## Abstract

**Aim:**

Type 2 Diabetes Mellitus (T2DM) is associated with microvascular complications, including diabetic retinopathy (DR), diabetic nephropathy (DNp), and diabetic peripheral neuropathy (DPN). In this study, we investigated genetic variations and Single Nucleotide Polymorphisms (SNPs) associated with DR, DNp, DPN and their combinations among T2DM patients of Arab origin from the United Arab Emirates, to establish the role of genes in the progression of microvascular diabetes complications.

**Methods:**

A total of 158 Emirati patients with T2DM were recruited in this study. The study population was divided into 8 groups based on the presence of single, dual, or all three complications. SNPs were selected for association analyses through a search of publicly available databases, specifically genome-wide association study (GWAS) catalog, infinome genome interpretation platform, and GWAS Central database. A multivariate logistic regression analysis and association test were performed to evaluate the association between 83 SNPs and DR, DNp, DPN, and their combinations.

**Results:**

Eighty-three SNPs were identified as being associated with T2DM and 18 SNPs had significant associations to one or more diabetes complications. The most strongly significant association for DR was rs3024997 SNP in the *VEGFA* gene. The top-ranked SNP for DPN was rs4496877 in the *NOS3* gene. A trend towards association was detected at rs833068 and rs3024998 in the *VEGFA* gene with DR and rs743507 and rs1808593 in the *NOS3* gene with DNp. For dual complications, the rs833061, rs833068 and rs3024997 in the *VEGFA* gene and the rs4149263 SNP in the *ABCA1* gene were also with borderline association with DR/DNp and DPN/DNp, respectively. Diabetic with all of the complications was significantly associated with rs2230806 in the *ABCA1* gene. In addition, the highly associated SNPs rs3024997 of the *VEGFA* gene and rs4496877 of the *NOS3* gene were linked to DR and DPN after adjusting for the effects of other associated markers, respectively.

**Conclusions:**

The present study reports associations of different genetic polymorphisms with microvascular complications and their combinations in Emirati T2DM patients, reporting new associations, and corroborating previous findings. Of interest is that some SNPs/genes were only present if multiple comorbidities were present and not associated with any single complication.

## Introduction

Type 2 Diabetes Mellitus (T2DM) is a major worldwide public health problem that currently affects over 425 million people globally, and by 2045 is expected to reach 628 million. By 2030 T2DM is projected to be the 7^th^ most common cause of death in the world (1, 2). In the Middle East and North Africa region alone, 9.6% of adults are living with T2DM, with approximately 49% undiagnosed. Furthermore, the Gulf region has one of the highest rates of T2DM in the world, with the United Arab Emirates (UAE) specifically having a prevalence of 23%, with 6.6%-14.6% of the population undiagnosed (3–5). Those numbers show the significant role that T2DM plays and the challenges it brings in health care and quality of life in the UAE.

People with diabetes have a higher risk of developing serious complications affecting all organ systems that increase medical costs and lower quality of life (6). In 60-70% of T2DM patients, diabetes is coupled with retinopathy (7), nephropathy (8), or peripheral neuropathy (9), which are ultimately the main cause of morbidity and mortality. The precise mechanism by which those complications occur is not completely understood; however, they most likely are multifactorial, including both environmental and genetic components (6, 10).

Nephropathy, peripheral neuropathy, and retinopathy, all affect the microvasculature. Diabetic nephropathy (DN) is a major cause of death in patients with diabetes that affects kidney filtration due to damaged blood vessels, causing a decrease in glomerular filtration rate (GFR) and an increase in microalbuminurea levels (11). Diabetic peripheral neuropathy (DPN) is one of the most common complications occurring in 50% of patients with diabetes and is characterized by peripheral nerve damage due to persistently high blood glucose levels (12). It starts in the lower limbs but can progress to upper limbs if unresolved (glove and stocking syndrome). It leads to loss of sensation, muscle weakness, numbness, and often, requires amputation of toes or parts of the lower limbs (13–16). Diabetic retinopathy (DR), on the other hand, affects retinal blood vessels, leading to mild symptoms such as blurry vision and, in severe cases, complete blindness (7, 17). Those complications do not always occur on their own but can be found in combination. The onset of diabetes, age of the patient as well as other factors, contribute to the progression of complications (18, 19). The best treatment options for diabetic complications are exercising, strict glucose, lipids and blood pressure control that possibly slow disease progression but not stopping it (9, 16, 20). Progression of diabetic complications are difficult to identify and quantify, especially at the asymptromatic stage. Therefore, to better understand diabetes and its complications, research needs to focus on genes, gene polymorphisms, and how these genes function that leads to diabetic complications. Numerous studies link specific genes to T2DM and its comorbidities, with many being associated with glucose metabolism and biochemical changes such as inflammation (7, 21, 22). Witzel et al. highlighted in a review paper that in patients with DPN the Angiotensin I Converting Enzyme (*ACE*) gene is significantly associated with Caucasian and Asian populations, whereas another study in an Indian population found the Nitric Oxide Synthase 3 (*NOS3*) gene to be highly associated with DPN. A third study in a Romanian population reported that Vascular Endothelial Growth Factor (*VEGF*) is significantly associated with DPN (16, 23, 24). Furthermore, Freedman et al. reported that the *CNDP1, ELMO1*, and *NOS3* genes were highly associated with DNp (8).

Investigation of the associations of these genes or SNPs with diabetic complications may provide a more focused approach to early detection, personalized medicine, and management of complications. However, the genes associated with diabetes and especially with diabetes complications, vary across different populations and studies (25, 26). For instance, in patients with DR, one study found that *VEGF* gene was significantly associated with a Japanese population but not in a Caucasian population (27, 28). Hence further research is required into the identification of genetic associations with diabetic complications in homogeneous populations with diabetes and with or without complications

This study aimed at filling this gap by identifying genes and polymorphisms that are significantly associated with T2DM and three microvascular complications in an Emirati cohort. The T2DM associated complications were focused on DPN, DNp, and DR as given their high rate of occurrence in T2DM patients. In addition, previous work by Witzel et al. (16), Jelinek et al. (18) and Azzam et al. (29) reported DR as the most frequent T2DM complication (13.26%) and DPN and DNp as the most common combined complication (8.57%) in the Emirati population.

## Methods

### Subjects

A total of 158 (110 females and 48 males), unrelated T2DM patients who were enrolled at Sheikh Khalifa Medical Center in Abu Dhabi, UAE, participated in the study between July 2014 and May 2015. All subjects were born in the UAE and of Arab descent. The demographic and clinical data are presented in **Table 1**.

**Table 1:** Demographic, clinical and laboratory characteristics of T2DM with and without various complication combinations.

Parameters		T2DM with no complication	Peripheral Neuropathy	Nephropathy	Retinopathy	Peripheral Neuropathy/Nephropathy	Retinopathy/Peripheral Neuropathy	Retinopathy/Nephropathy	Retinopathy/Peripheral Neuropathy/Nephropathy	p- value
*N*		*32*	*14*	*10*	*35*	*4*	*26*	*20*	*17*	
*Gender*	*Female (n=110)*	*20* *12*	*9* *5*	*6* *4*	*29* *6*	*3* *1*	*20* *6*	*14* *6*	*9* *8*	*0.404*
	*Male (n=48)*									
*Age (years)*		*52.21± 2.00*	*52.07± 3.44*	*62.5± 4.59*	*57.94± 1.55*	*59± 2.48*	*59.92± 1.96*	*63.05± 2.39*	*65.41± 3.49*	0.001*
*BMI (kg/m^2^)*		*33.33± 1.30*	*32.61± 1.79*	*31.13± 1.47*	*34.14± 1.13*	*31.62± 0.86*	*33.02± 1.18*	*33.41± 1.38*	*30.81± 1.26*	*0.739*
*Smoking*	*Current (n=13)*	*3*	*3* *3* *8* *8*	*1* *1* *8*	*1* *3* *31*	*0* *1* *3*	*3* *3* *20*	*2* *1* *17*	*0* *4* *13*	*0.593*
	*Previous (n=21)*	*4*								
	*Never (n=124)*	*24*								
*Hypertension*		*19*	*11*	*8*	*24*	*4*	*19*	*15*	*19*	*0.117*
*Systolic blood pressure (mmHg)*		*130.40± 2.95*	*124.71± 3.61*	*125.30± 5.52*	*127.57± 2.05*	*137.25± 8.68*	*127.42± 3.16*	*136.65± 3.04*	*133.23± 4.25*	*0.211*
*Diastolic blood pressure (mmHg)*		*80.25± 1.37*	*76.71± 2.61*	*72.90± 3.51*	*78.25± 1.48*	*83.25± 0.47*	*74.46± 2.40*	*81.35± 2.07*	*75.05± 3.28*	*0.100*
*Waist circumference (cm)*		*105.06± 2.80*	*107.96± 2.10*	*100.90± 4.85*	*106.14± 1.86*	*107.75± 4.38*	*105.67± 2.30*	*107.44± 2.78*	*108.13± 3.31*	*0.894*
*HbA1c (%)*		*8.23± 0.41*	*8.01± 0.41*	*7.62± 0.41*	*7.98± 0.31*	*8.33± 1.01*	*7.70± 0.29*	*7.54± 0.31*	*7.86± 0.39*	*0.884*
*Fasting blood glucose (mmol/l)*		*9.07± 1.47*	*7.84± 0.88*	*9.04± 0.83*	*10.35± 0.79*	*12.50± 4.50*	*10.09± 1.05*	*7.80± 0.61*	*8.63± 1.01*	*0.383*
*Dyslipidemia (n=148)*		*22*	*14*	*10*	*35*	*4*	*26*	*20*	*17*	0.003*
*Triglyceride (mmol/l)*		*1.50± 0.17*	*1.60± 0.19*	*1.58± 0.42*	*1.41± 0.13*	*0.96± 0.12*	*1.71± 0.25*	*1.75± 0.22*	*1.35± 0.16*	*0.736*
*Total cholesterol (mmol/l)*		*4.62± 0.26*	*3.76± 0.29*	*4.32± 0.51*	*4.05± 0.19*	*3.87± 0.63*	*3.79± 0.18*	*3.87± 0.21*	*3.73± 0.27*	*0.172*
*HDL cholesterol (mmol/l)*		*1.30± 0.09*	*1.06± 0.11*	*1.14± 0.12*	*1.35± 0.07*	*1.20± 0.24*	*1.43± 0.19*	*1.13± 0.05*	*1.23± 0.07*	*0.466*
*LDL cholesterol (mmol/l)*		*2.59± 0.24*	*2.07± 0.24*	*2.19± 0.37*	*2.10± 0.15*	*2.23± 0.37*	*1.71± 0.15*	*1.93± 0.18*	*1.88± 0.25*	*0.096*
*Microalbumin (mg)*		*12.95± 5.65*	*13.50± 6.12*	*20.40± 14.77*	*28.94± 13.66*	*-*	*65.26± 36.89*	*101.66± 47.03*	*21.10± 8.46*	*0.518*
*Creatinine (µmol/l)*		*58.60± 3.37*	*64.85± 4.41*	*133.55± 18.51*	*57.33± 2.72*	*273.0± 160.50*	*69.70± 3.31*	*106.52± 12.84*	*84.50± 7.28*	<0.001*
*Urea (mmol/l)*		*4.37± 0.28*	*5.00± 0.42*	*7.17± 1.06*	*4.16± 0.24*	*12.23± 5.43*	*4.84± 0.31*	*6.55± 0.65*	*6.50± 0.67*	<0.001*
*eGFR (ml/min)*		*106.43± 3.55*	*100.85± 5.47*	*51.44± 9.73*	*99.03± 2.46*	*42.75± 12.77*	*87.19± 2.92*	*65.20± 6.30*	*73.03± 7.07*	<0.001*
*Serum 25(OH)D (nmol/l)*		*60.90± 6.79*	*69.26± 11.73*	*69.28± 12.02*	*67.07± 4.79*	*45.25± 12.25*	*67.42± 5.91*	*58.22± 4.91*	*73.95± 7.87*	*0.705*

*P value for < 0.05; All continuous data is shown as mean ± SD, calculated using a two-sided t-test. P value for percentage data calculated using the Pearson χ2 test. BMI, body mass index; eGFR, estimated glomerular filtration rate; HDL, high-density lipoprotein; LDL, low-density lipoprotein; N, number of individuals; T2DM, type 2 diabetes mellitus. Bold values reaching the significant level with a p value < 0.05.

Patients were recruited to this study if they were UAE born nationals, diagnosed with T2DM and above 18 years old at the time of the study. Participants agreed to take part in this study after a briefing session and upon signing an informed consent form that had been approved by the Institutional Ethics Committee of the hospital (Sheikh Khalifa Medical Center-IRB-Ref R292). However, patients were excluded if unable to give consent, pregnant women, or any pathophysiology disease such as cancer or psychosis was present.

### Diagnosis of T2DM and Complications

The patients included in the study were all diagnosed with T2DM either from the medical records or reported use of medication at the time of the hospital data collection. The presence of diabetic associated complications was confirmed by a qualified physician, based on the criteria outlined by the World Health Organization (WHO) consultation group report (30).

Diagnosis of peripheral neuropathy was based on the presence of foot ulcers, loss of sensation or numbing in the feet, loss of a toe, foot or leg due to diabetes, pain in calf muscles while walking, or diagnosis of peripheral vascular disease in the legs. The presence of nephropathy was determined by urine albumin level and set at higher than 20 μg/min for microalbuminuria and higher than 200 μg/min for macroalbuminuria or if the estimated glomerular filtration rate (eGFR) was less than 60 ml/min/1.73m^2^. Retinopathy was defined as either white or red lesions or both being present in the retina according to WHO criteria (31).

This study was divided into 8 patient groups: 1) a control group, which included patients with diabetes and no complications, 2) DPN: patients with diabetic peripheral neuropathy, 3) DR: patients with diabetic retinopathy, 4) DNp: patients with diabetic nephropathy, 5) DR/DNp: patients with retinopathy and nephropathy, 6) DPN/DNp: patients with peripheral neuropathy and nephropathy, 7) DR/DPN: patients with retinopathy and peripheral neuropathy, and 8) DR/DPN/DNp: patients with retinopathy, nephropathy, and peripheral neuropathy

### Clinical Variables and Laboratory Data

Laboratory tests were performed at the time of enrollment. Blood pressure was taken on two different occasions, and the average was taken. Hypertension was defined as systolic blood pressure >130 mm Hg, diastolic blood pressure >80 mm Hg, or taking antihypertensive medications. Dyslipidemia was diagnosed as a total cholesterol >6.2 mmol/L, low-density lipoprotein (LDL)-cholesterol >3.3 mmol/L, and or triglycerides >2.2 mmol/L. A calibrated wall-mounted stadiometer and weight scale were used by trained nurses to measure height and weight, respectively, which were then used to calculate the body mass index (BMI) as the weight in kilograms divided by the square of the height in meters.

### Sample Collection and DNA Extraction

Approximately two milliliters of saliva was collected from each study subject using the Oragene OGR-500 kit (DNA Genotek, Ottawa, Canada). Genomic DNA from the buccal cells in these saliva samples were extracted using the prepIT^®^ L2P system (DNA Genotek, Ottawa, Canada) according to the manufacturer protocol. Each aliquot of extracted DNA was quantified using the Nanodrop 2000C Spectrophotometer (Thermo Scientific, Wilmington, USA).

### Genotyping

Genotyping was performed following the manufacturer’s protocols on the Infinium Omni5ExomeHuman chip (Illumina Inc., San Diego, USA), and raw data was collected on the GenomeStudio v2010.3 (Illumina Inc., San Diego, USA). The microarray contained 4,641,218 SNPs. Quality control on the data was performed using PLINK software (version 1.07) (32) to remove SNPs with a minor allele frequency (MAF) <0.05, with >5% missing genotype rate, failing the Hardy-Weinberg equilibrium (HWE) test at the 0.000001 significance level and Mendelian error. Approximately 39% of SNPs passed these Quality Control criteria.

### SNP Selection

The SNPs were selected after a search in multiple resources associated with T2DM, DPN, DNp and DR. The search engines and databases utilized include Google Scholar, PubMed, the Genome Wide Association Studies (GWAS) catalog (https://www.ebi.ac.uk/gwas/home), the infinome genome interpretation platform (https://www.infino.me/) and the GWAS Central database (http://www.gwascentral.org/). All SNPs located within genes that have been previously reported in association with T2DM in different ethnic groups and the complications were included. In total, 83 genetic loci were identified as linked to DPN, DNp, DR, or a combination of these.

### Statistical Analysis

Statistical analyses for all demographic, clinical, and laboratory variables were performed using Stata software V.14. Continuous data is reported as mean ± SD; statistical differences were assessed using two-sided t-tests for normally distributed data or for highly skewed data the Wilcoxon rank-sum (Mann-Whitney) test was utilized. The Pearson χ2 test was used for percentage data unless the expected frequencies were less than 5, in which case Fisher’s exact test was implemented. A p value<0.05 was considered significant.

The genotype frequencies were tested for HWE using the HWE Institute of Human Genetics calculator https://ihg.gsf.de/ihg/index_engl.html through the Pearson χ^2^ test for T2DM with no complication and T2DM with complications. The “SNPassoc” package of the R version 3.3.1 (R Foundation, Vienna, Austria) was used to evaluate the association between a T2DM complication as a result (response or dependent variable) and the presence of a SNP (predictor or independent variable) through multivariate logistic regression analysis and association test. The p-values are adjusted for age and gender and were tested for dominant and additive genetic models. The Bonferroni correction was performed to account for multiple testing that is conducted in this study, where the α-value is adjusted from 0.05 to a new α-value = (0.05/N), and *N* refers to the number of statistical test performed. In the current study, 18 SNPs were investigated in association with seven T2DM complications. Therefore, a new α-value = (0.05/18 x 7) = 0.0004 wasset. Results from our multiple testing approach describe a complication as significantly associated SNP when *p* < 0.0004. Gene-Gene interactions of the top associated SNPs were investigated using a multiple logistic regression model in R software. The protein-protein interaction network and gene pathway interaction were constructed using the Gene Multiple Association Network Integration Algorithm (GeneMANIA, http://www.genemania.org/) (33). The chi-squared test was performed to calculate the differences in allelic frequencies of the most associated SNPs between the current study (Arab, UAE) and other ethnic groups (European, African, East Asian, Ashkenazi Jewish, and American). The allele frequencies data for the ethnic groups of interest were taken from the gnomAD database (34), and the American group consists of Latino/Admixed Americans.

## Results

### Characteristics of Study Subjects

This study included a group size of 158 T2DM patients, 32 of whom had no complications, and 126 with either retinopathy, nephropathy, peripheral neuropathy, or a combination of these. Patients were categorized into 8 groups according to the T2DM complications they hold. **Table 1** summarizes the demographic, clinical, and laboratory characteristics of the different patient groups in this study.

A significant difference in age was observed between the groups with patients with no complications the youngest (52.21± 2.00 yrs) followed by those with single complications (52.07± 3.44 – 62.5± 4.59 yrs), dual complications (59± 2.48 - 63.05± 2.39 yrs) and patients with all three complications in this study being the oldest (65.41± 3.49 yrs). Most of the patients were female and accounting for more than twice that of males. Dyslipidemia was present in all patients with complications and in 69% of the no complications group, making it a highly significant cofactor in disease progression. All groups presented high HbA1c and fasting blood glucose levels but the lipids profile for all groups were within the expected range. Microalbumin levels were much higher in patients with DR/DPN and DR/DNp, but no significance was detected compared to diabetics with no complications. However significant differences between groups were found for the creatinine, urea and eGFR values, where patients with nephropathy whether alone (DNp) or in combined with either neuropathy or retinopathy (DPN/DNp, DR/DNp) had significantly higher levels of creatinine, urea, and lower eGFR levels compared to other single complications or combinations of complications, which marks a clear decline in kidney function.

### Association Between SNPs and T2DM Complication

The genotype, allele frequencies and HWE test results for the investigated SNPs are detailed in **Supplementary Table 1**. HWE was examined for the 18 SNPs in the T2DM with no complication and T2DM with complications groups, indicating that no deviation from HWE were identified (p-value< 0.01).

**Figure 1:**
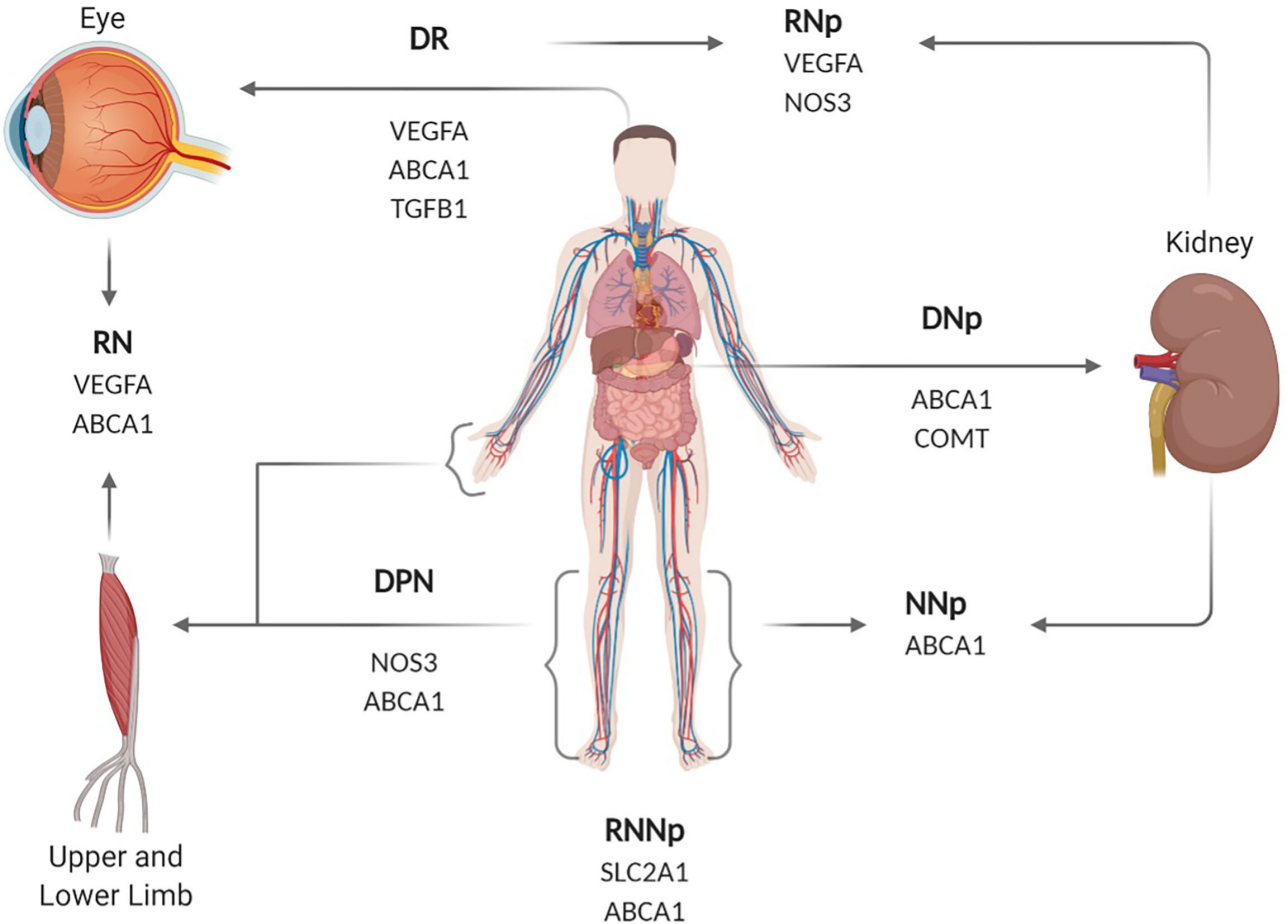
Diagram showing the genes associated with the different complications, highlighting which area of the human body they affect. DPN, diabetic peripheral neuropathy; DNp, diabetic nephropathy; DR, diabetic retinopathy; DPN/DNp, diabetic peripheral neuropathy and nephropathy; DR/DPN, diabetic retinopathy and peripheral neuropathy, DR/DNp, diabetic retinopathy and nephropathy; DR/DPN/DNp, diabetic retinopathy, peripheral neuropathy and nephropathy. SLC2A1, Solute Carrier Family 2 Member 1; VEGFA, Vascular endothelial growth factor A; NOS3, Nitric Oxide Synthase 3; ABCA1, ATP Binding Cassette Subfamily A Member 1; TGFB1, Transforming Growth Factor Beta 1; COMT, Catechol-O-methyltransferase. Created with BioRender.com.

**Figure 2:**
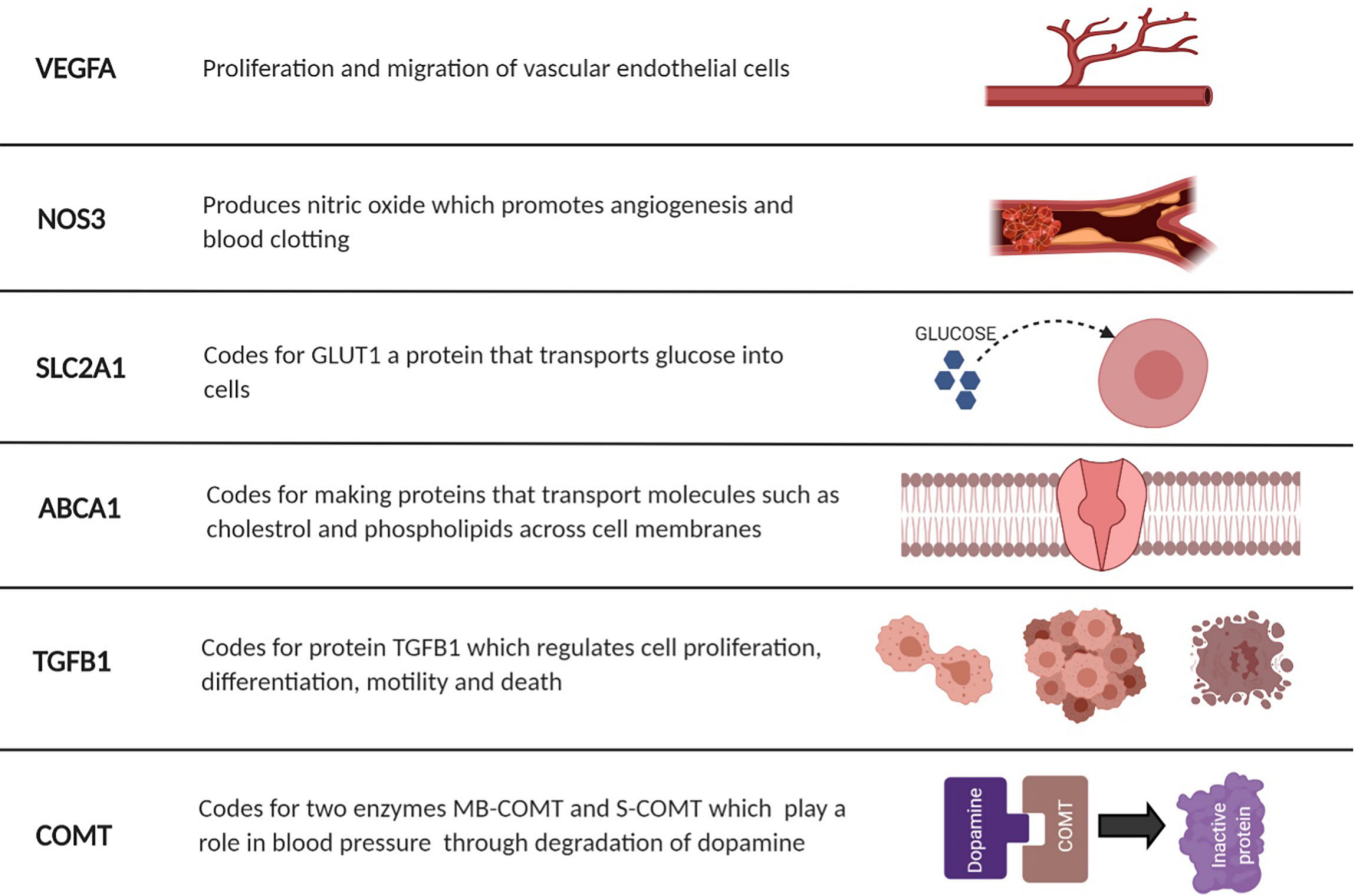
Illustrative figure summarizing the role each of gene and potential complications. SLC2A1, Solute Carrier Family 2 Member 1; VEGFA, Vascular endothelial growth factor A; NOS3, Nitric Oxide Synthase 3; ABCA1, ATP Binding Cassette Subfamily A Member 1; TGFB1, Transforming Growth Factor Beta 1; COMT, Catechol-O-methyltransferase. Created with BioRender.com.

The analysis of 83 SNPs associated with T2DM and its complications led to finding 18 SNPs with significant associations to one or more complication (p-value < 0.05). **Table 2** summarizes the results of the significant genetic SNPs associated with the various complications in the T2DM patient groups. Three SNPs in *VEGFA* gene showed an association with DR in at least one of the two analysis tested for the association: rs3024997 highly associated with DR (p-value= 0.0008) and rs833068 (p-value= 0.002) and rs3024998 (p-value= 0.005) with limited association with DR. In the case of DNp, rs743507 and rs1808593 (p-value= 0.004) in the *NOS3* gene were found to be as borderline significant (p-value < 0.01). The rs4496877 SNP in NOS3 gene were found to be highly associated with DPN (p-value = 0.0004). For dual complications the rs833061 (p-value= 0.004), rs833068 (p-value= 0.005) and rs3024997 (p-value= 0.001) *VEGFA* SNPs were also with borderline association with DR/DNp. Furthermore, the SNP rs4149263 (*ABCA1*) (p-value= 0.006) were significantly associated with DPN/DNp. Diabetic with all of the complications was significantly associated with rs2230806 in the *ABCA1* gene (p-value= 0.008). **Figure 1** illustrates the genes, their associated complication(s) and the part of the body they affect. **Figure 2** illustrates the role of each gene in potential complications.

**Table 2:** Significant genetic SNP associations to the various complications in the T2DM patient group.

Gene	Chr	SNPs	BP	Locus relative to gene	Retinopathy		Nephropathy		Peripheral neuropathy		Retinopathy/Nephropathy		Retinopathy/Peripheral Neuropathy		Peripheral Neuropathy/Nephropathy		Retinopathy/Peripheral Neuropathy/Nephropathy	
					“L” p-value Adjusted	“A” p-value Adjusted	“L” p-value Adjusted	“A” p-value Adjusted	“L” p-value Adjusted	“A” p-value Adjusted	“L” p-value Adjusted	“A” p-value Adjusted	“L” p-value Adjusted	“A” p-value Adjusted	“L” p-value Adjusted	“A” p-value Adjusted	“L” p-value Adjusted	“A” p-value Adjusted
**SLC2A1**	**1**	rs841853_T	42935767	Intron Variant	0.578	0.457	0.715	0.044*	0.231	0.340	0.452	0.078	0.768	0.173	0.433	0.232	0.033*	0.268
**VEGFA**	**6**	rs833061_C	43769749	Upstream gene variant	0.016*	–	0.478	–	0.168	–	0.004**	–	0.369	–	0.592	–	0.89	–
		rs833068_A	43774790	Intron Variant	0.002**	0.004**	0.492	0.185	0.458	0.888	0.005**	0.010*	0.0842	0.180	0.605	0.825	0.437	0.190
		rs3024997_A	43777370	Intron Variant	0.0008***	0.001**	0.262	0.071	0.613	0.633	0.001**	0.028*	0.0186*	0.167	0.981	0.600	0.465	0.057
		rs3024998_T	43777840	Intron Variant	0.005**	0.022*	0.517	0.435	0.5814	0.785	0.012*	0.143	0.0503	0.428	0.704	0.676	0.625	0.112
**NOS3**	**7**	rs4496877_T	150983418	Unknown	0.43	0.579	0.149	0.213	0.119	0.0004***	0.037*	0.989	0.473	0.423	0.469	0.302	0.884	0.829
		rs743507_G	151010400	Intron variant	0.511	0.994	0.272	0.004**	0.034*	–	0.194	0.259	0.227	–	0.948	–	0.0699	–
		rs1808593_G	151011214	Intron variant	0.533	0.994	0.372	0.004**	0.032*	–	0.223	0.327	0.200	–	0.864	–	0.0809	–
**ABCA1**	**9**	rs4149339_T	104782875	3’ UTR Variant	0.0588	0.316	0.238	–	0.692	0.345	0.117	0.045*	0.0351*	0.211	0.25	0.704	0.711	0.082
		rs2020927_C	104790904	Intron variant	0.735	0.893	0.039*	0.632	0.806	0.387	0.277	0.883	0.987	0.585	0.57	0.314	0.936	0.352
		rs2230806_A	104858586	Missense Variant	0.077	0.350	0.693	0.937	0.057	0.357	0.123	0.321	0.206	0.233	0.267	0.598	0.008**	0.020*
		rs2249891_G	104861961	Intron variant	0.049*	0.359	0.665	–	0.285	–	0.224	–	0.058	0.361	0.706	–	0.172	0.381
		rs4149268_G	104884939	Intron variant	0.109	0.857	0.269	0.368	0.175	0.991	0.166	0.682	0.206	0.353	0.503	0.614	0.05*	0.013*
		rs3905000_A	104894789	Intron variant	0.46	–	0.028*	–	0.036*	–	0.224	–	0.518	0.323	0.019*	–	0.017*	0.031*
		rs4149263_C	104915008	Intron variant	0.976	0.196	0.032*	0.052	0.573	0.061	0.13	0.164	0.197	0.593	0.106	0.006**	0.497	0.400
**TGFB1**	**19**	rs1800469_T	41354391	Upstream gene variant	0.011*	0.257	0.347	0.422	0.378	0.625	0.147	0.772	0.066	0.429	0.252	0.375	0.991	0.807
		rs4803457_T	41355454	Upstream gene variant	0.017*	0.282	0.813	0.811	0.228	0.838	0.119	0.754	0.22	0.735	0.279	0.716	0.933	0.382
**COMT**	**22**	rs933271_C	19943884	Intron variant	0.918	0.174	0.045*	0.078	0.165	0.039*	0.725	0.271	0.267	0.511	0.131	0.062	0.731	0.014*

*P value for < 0.05; **P < 0.01; ***P <0.001. BP, base pair position; Chr, chromosome; SNP, single nucleotide polymorphism; T2DM, type 2 diabetes mellitus; SLC2A1, Solute Carrier Family 2 Member 1; VEGFA, Vascular endothelial growth factor A; NOS3, Nitric Oxide Synthase 3; ABCA1, ATP Binding Cassette Subfamily A Member 1; TGFB1, Transforming Growth Factor Beta 1; COMT, Catechol-O-methyltransferase. “L” p-value for multivariate logistic regression analysis adjusted for Age and Gender. “A” p-value for the association of the SNPs with the various complications in T2DM patient group adjusted for age and gender were tested for dominant model (AA vs Aa+aa). Bold values reaching the significant level with a p value < 0.05.

### Gene-Gene Interaction Analyses

To test the combined effects of the genes related to the SNPs associated with diabetic complications in this study, we performed a Gene-Gene interaction analysis, as shown in **Table 3**, using an additive model. After adjusting for the effects of other associated markers, the highly associated SNPs rs3024997 of the VEGFA gene and rs4496877 of the NOS3 gene were linked to DR and DPN, respectively (p-value= 0.044 and 0.022). In addition, the rs3905000 and rs4149263 of *ABCA1* gene were associated with DNp and DPN/DNp, respectively without the effect of other associated SNPs (p-value= 0.024 and 0.044). The analysis revealed an interaction between the *NOS3* rs743507 SNP located on chromosome 7 and the ABCA1 rs3905000, rs2020927 (p-value = 0.009) or rs4149263 (p-value = 0.022) SNPs located on chromosome 9. This result suggests that the genotype of the markers rs743507, rs2020927 and rs4149263 may have a modifying effect on the risk of DNp instigated by marker rs3905000. A significant effect of gene-gene interaction in DR/DNp with a p-value of 0.041 was found for rs833068, rs3024997 of the *VEGFA* gene and rs4496877 of the *NOS3* gene. Finally, the three SNPs rs2230806, rs4149268 and rs3905000 of *ABCA1* gene have overall effects with the susceptibility of all three diabetic complications.

**Table 3:** Effects of gene-gene interaction of the top associated SNPs on the risk of diabetic complications..

Complications	SNPs (Genes)	Estimate (Beta)	SE	*p-*value
**Retinopathy**	rs833061_C (*VEGFA*)	0.038	0.142	0.788
	rs833068_A (*VEGFA*)	-0.576	0.901	0.525
	rs3024997_A (*VEGFA*)	1.508	0.731	0.044*
	rs3024998_T (*VEGFA*)	-0.155	0.90	0.864
	rs841853_T (*SLC2A1*)	-9.773e-16	3.311e-01	1.000
**Nephropathy**	rs743507_G (*NOS3*)	1.250e-01	5.300e-01	0.817
	rs1808593_G (*NOS3*)	NA	NA	NA
	rs2020927_C (*ABCA1*)	-1.124e-16	2.137e-01	1.000
	rs3905000_A (*ABCA1*)	1.000e+00	3.941e-01	0.024*
	rs4149263_C (*ABCA1*)	-1.514e-15	3.941e-01	1.000
	rs743507_G:rs3905000_A	-2.750e+00	1.081e+00	0.024*
	rs743507_G:rs2020927_C:rs3905000_A	2.688e+00	8.808e-01	0.009**
	rs743507_G:rs2020927_C:rs4149263_C	-1.438e+00	6.078e-01	0.034*
	rs743507_G:rs3905000_A:rs4149263_C	2.750e+00	1.059e+00	0.022*
**Peripheral neuropathy**	rs4496877_T (*NOS3*)	-0.454	0.191	0.022*
	rs743507_G (*NOS3*)	-0.077	0.164	0.641
	rs1808593_G (*NOS3*)	NA	NA	NA
**Retinopathy/nephropathy**	rs833061_C (*VEGFA*)	-0.290	0.257	0.267
	rs833068_A (*VEGFA*)	0.372	0.603	0.541
	rs3024997_A (*VEGFA*)	-2.026	1.358	0.144
	rs3024998_T (*VEGFA*)	1.079	1.003	0.289
	rs4496877_T (*NOS3*)	-0.406	0.417	0.336
	rs833068_A:rs3024997_A:rs4496877_T	-1.020	0.481	0.041*
**Peripheral neuropathy/nephropathy**	rs3905000_A (*ABCA1*)	-3.846e-02	1.285e-01	0.7667
	rs4149263_C (*ABCA1*)	2.500e-01	1.196e-01	0.044*
	rs3905000_A:rs4149263_C	1.538e-01	1.745e-01	0.384
**Retinopathy/Peripheral neuropathy/nephropathy**	rs841853_T (*SLC2A1*)	0.009	0.474	0.984
	rs2230806_A (*ABCA1*)	-0.077	0.442	0.863
	rs4149268_G (*ABCA1*)	-0.047	0.294	0.872
	rs3905000_A (*ABCA1*)	-7.043	3.411	0.052
	rs933271_C (COMT)	2.212	2.037	0.290
	rs2230806_A:rs4149268_G:rs3905000_A	-2.611	0.932	0.011*

Values in bold indicate significant *p-value for < 0.05. SNP, single nucleotide polymorphism; Beta, regression coefficient for interaction in logistic regression; SE, standard error; SLC2A1, Solute Carrier Family 2 Member 1; VEGFA, Vascular endothelial growth factor A; NOS3, Nitric Oxide Synthase 3; ABCA1, ATP Binding Cassette Subfamily A Member 1; COMT, Catechol-O-methyltransferase.

**Figure 3:**
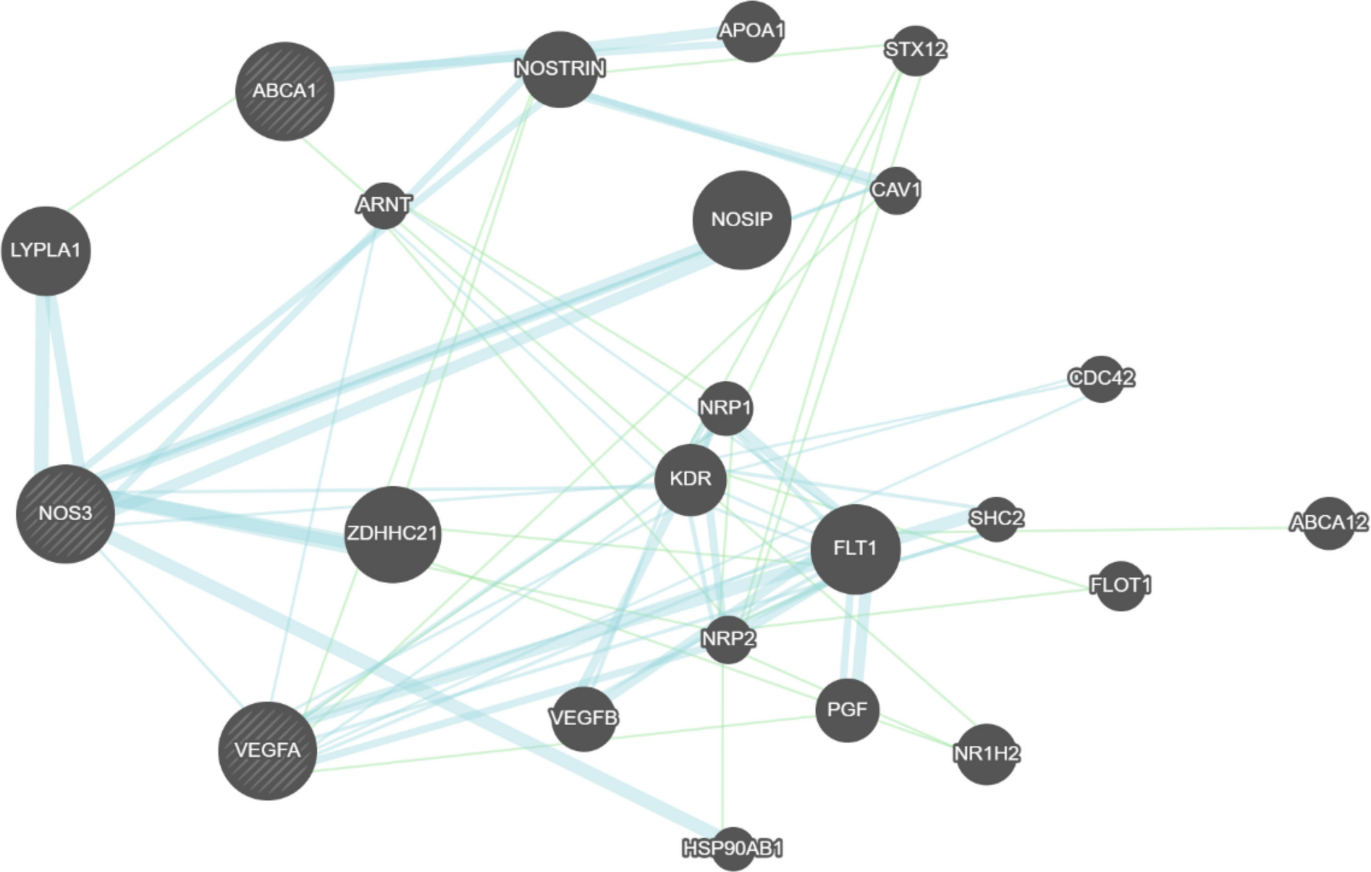
Constructed interaction networks including all the genes related to the top SNPs associated with diabetic complications in this study. Black circles indicate the queried genes. Green lines represent the genetic interactions. Blue lines represent the protein-protein interaction.

The gene pathway interaction and the protein-protein interaction network between the SNP-linked genes investigated in this study were explored in **Figure 3** to elucidate the potential regulatory mechanism of diabetic complications. The results of GeneMANIA software indicate a direct protein-protein interaction between VEGFA and NOS3 and indirect gene-gene interaction between *ABCA1* and *NOS3* genes which validate our previous result of gene-gene interaction.

### Comparaison of Allele Frequencies and Association Studies Between UAE and Other Ethnic Groups

A thorough literature search was performed to compare the current allele frequencies and genetic associations of the top associated SNPs to those of previous studies conducted among different populations. The results recorded in **Table 4** give a greater insight on the similarity and differences between the Emirati population’s allele frequency data compared to other ethnic groups such as European, African, East Asian, Ashkenazi Jewish, and American in the context of diabetic complications. The ethnic group that was found to have the highest number of SNPs with a significantly different allelic frequency from the Emirati population is the European ethnic group. Moreover, 5 out of the 9 top associated SNPs differed significantly in the allele frequencies between the European population and the Emirati population in our study. On the basis of chi-squared test, the comparison of our data with other ethnic groups showed significant differentiation ranked from the greatest number of SNPs to the least (European differed at 5, African and East Asian differed at 3, American differed at 2 and Ashkenazi Jewish differed at 1 out of the 9 top significant SNPs of this study).

**Table 4:** Allele frequencies of SNPs most strongly associated with various complications in our population (Arab) and other ethnicities.

Gene	Chr : BP	SNP	Population	N	Allele frequency (%)		*p*-value
					T	C	
*VEGFA*	6:43769749	rs833061	Arab (UAE)	157	39.1	60.9	Ref
			European	7454	51.4	48.6	0.0024**
			African	5684	34.5	65.5	0.295
			East Asian	1159	25.0	75.0	0.0003***
			Ashkenazi Jewish	162	44.2	55.8	0.368
			American	504	40.2	59.8	0.842
					**G**	**A**	
*VEGFA*	6:43774790	rs833068	Arab (UAE)	157	59.9	40.1	Ref
			European	4481	70.9	29.1	0.004**
			African	4052	53.2	46.8	0.117
			East Asian	649	58.1	41.9	0.767
			Ashkenazi Jewish	132	54.5	45.5	0.427
			American	325	61.6	38.4	0.801
					**G**	**A**	
*VEGFA*	6:43777370	rs3024997	Arab (UAE)	158	62.4	37.6	Ref
			European	4438	71.2	28.8	0.025*
			African	2755	68.3	31.7	0.163
			East Asian	656	57.7	42.3	0.287
			Ashkenazi Jewish	131	54.9	45.1	0.228
			American	314	63.0	37.0	1.000
					**C**	**T**	
*VEGFA*	6:43777840	rs3024998	Arab (UAE)	158	64	36.0	Ref
			European	4429	71.2	28.8	0.059
			African	2838	67.2	32.8	0.444
			East Asian	658	57.5	42.5	0.163
			Ashkenazi Jewish	131	54.9	45.1	0.153
			American	316	62.6	37.4	0.933
					**G**	**T**	
*NOS3*	7:150983418	rs4496877	Arab (UAE)	157	26.6	73.40	Ref
			European	9836	36.1	63.9	0.019*
			African	8151	6.4	93.6	< 2.2e-16***
			East Asian	1376	11.3	88.7	7.97e-08***
			Ashkenazi Jewish	192	33.8	66.2	0.188
			American	641	24.3	75.7	0.599
					**A**	**G**	
*NOS3*	7:151010400	rs743507	Arab (UAE)	156	21.6	78.40	Ref
			European	11953	22.4	77.6	0.934
			African	6640	23.8	76.2	0.627
			East Asian	1192	23.6	76.4	0.694
			Ashkenazi Jewish	208	28.3	71.7	0.193
			American	704	16.8	83.2	0.169
					**T**	**G**	
*NOS3*	7:151011214	rs1808593	Arab (UAE)	158	21.3	78.7	Ref
			European	11912	22.5	77.5	0.843
			African	6636	23.6	76.4	0.607
			East Asian	1187	23.5	76.5	0.649
			Ashkenazi Jewish	208	28.3	71.7	0.171
			American	706	16.8	83.2	0.203
					**G**	**A**	
*ABCA1*	9:104858586	rs2230806	Arab (UAE)	158	55.4	44.6	Ref
			European	35425	72.6	27.4	2.308e-06***
			African	15592	37.5	62.5	3.956e-06***
			East Asian	8580	57.0	43.0	0.191
			Ashkenazi Jewish	2904	72.0	28.0	0.0003***
			American	11474	67.7	32.3	0.001*
					**T**	**C**	
*ABCA1*	9:104915008	rs4149263	Arab (UAE)	158	73.3	26.7	Ref
			European	3186	79.4	20.6	0.087
			African	1185	86.4	13.6	3.106e-05***
			East Asian	270	82.7	17.3	0.032*
			Ashkenazi Jewish	81	71.9	28.1	0.885
			American	131	84.6	15.4	0.028*

SNP, single nucleotide polymorphism; N, number of individuals; VEGFA, Vascular endothelial growth factor A; NOS3, Nitric Oxide Synthase 3; ABCA1, ATP Binding Cassette Subfamily A Member 1; Ref, reference study; UAE, United Arab Emiratis. *p-value for < 0.05; **p-value < 0.01; ***p-value <0.001 are considered statistically significant. Allele frequencies data of European, African, East Asian, Ashkenazi Jewish, and American ethnic groups are obtained from dbSNP (NCBI database of genetic variation) using gnomAD-Genomes study. In our population (Arab) the variability in number of participants is due to inability to obtain the genotyping results of few samples. Bold values reaching the significant level with a p value < 0.05.

**Table 5** summarizes the associations of the top associated SNPs with diabetic complications as reported in the literature among three different ethnic groups (Asians, Europeans and Middle Eastern).

**Table 5:** Comparison of association studies on diabetic complications between the UAE population and other ethnic groups.

SNPs	Genes	Population	OR [95% CI]	*p*-value	References
rs833061	*VEGFA*	Asian	2.12 [1.12-4.01]	0.02*	(35)
		European	2.24 [1.50-3.36]	<0.0001***	(36)
		Arab (UAE)	1.01 [1.00-1.02]	0.001**	
rs833068	*VEGFA*	Asian	–	0.035*	(37)
		European	3.1 [1.3-7.2]	0.017*	(38)
		Arab (UAE)	1.23 [1.05-1.42]	0.009**	
rs3024997	*VEGFA*	European	1.12 [0.88-1.44]	0.137	(39)
		Arab (UAE)	1.27 [1.09-1.47]	0.002**	Novel Retino
rs3024998	*VEGFA*	European	–	–	–
		Arab (UAE)	1.21 [1.04-1.41]	0.015*	Novel retino
rs4496877	*NOS3*	European	–	**-**	–
		Arab (UAE)	0.73 [0.59-0.91]	0.008**	Novel neuro
rs743507	*NOS3*	European	1.43 [1.03-2.00]	0.035*	(40)
		Arab (UAE)	1.41 [1.18-1.68]	0.0005***	
rs1808593	*NOS3*	European	–	0.700	(41)
		Arab (UAE)	1.41 [1.18-1.68]	0.0005***	
rs2230806	*ABCA1*	Asian	0.81 [0.08 - 7.57]	0.853	T2DM (42)
		Arab (UAE)	1.15 [0.97-1.37]	0.001**	Novel RNpPN
rs4149263	*ABCA1*	European	–	–	–
		Arab (UAE)	1.38 [1.15-1.66]	0.001**	Novel NpPN

*p-value for < 0.05; **p-value < 0.01; ***p-value <0.001 are considered statistically significant. SNP, single nucleotide polymorphism; Chr, chromosome; OR, odds ratio; CI, confidence intervals; UAE, United Arab Emirates; VEGFA, Vascular endothelial growth factor A; NOS3, Nitric Oxide Synthase 3; ABCA1, ATP Binding Cassette Subfamily A Member 1. ORs and 95% CI were reported with respect to the minor allele using an additive model in logistic regression adjusted for age and gender in UAE population. Bold values reaching the significant level with a p value < 0.05.

## Discussion

Numerous global studies report genetic associations with T2DM and its complications. However, the results vary widely and are inconclusive (43), possibly due to genetic differences in different family ancestries, which makes studying genetic associations in a specific cohort that much more important (44). The current research focused on an Emirati cohort where T2DM and its complications are highly prevalent, mainly attributed to the conserved gene pool and lifestyle factors leading to obesity, hypertension, and lipidemia (45, 46). It also adds to previous work published from the UAE by Azzam et al. (29) and Ossman et al. (47), which investigated genetic associations with diabetic retinopathy and diabetic kidney disease, respectively. However, the current study focused on different genes and polymorphisms and investigated diabetic peripheral neuropathy, retinopathy, and nephropathy, as well as their combinations in order to obtain a better understanding of the possible associations between the SNPs and microvascular complications of T2DM in the Emirati population.

### Demographic Data

Demographic and clinical data show that age is a major contributing risk factor for the development of diabetic complications. Younger patients had T2DM with single or no complications, conversely with increasing age combinations of diabetes becomes more common. This is in line with research findings by Jelinek et al. (18), where the reported prevalence of complications increased from 80% up to 94% in the older populations above 65 years. These findings may be attributed to decreased insulin sensitivity with age, which affects beta-cell functionality and increases insulin resistance (48). Furthermore, in patients with nephropathy as a single or dual comorbidity, creatinine and urea are significantly higher, and eGFR is much lower compared to the other complication groups. This is in line with previous studies since the aforementioned parameters act as biomarkers for kidney function and filtration, which are extremely affected by nephropathy due to blood vessel damage (49). In addition to biochemical markers, genotype and gene polymorphism may also play an important role in the presence of diabetic complications.

### Diabetic Retinopathy

DR is a major cause of visual impairment in elderly patients. It has an overall prevalence of 22-37% in individuals with known diabetes and leads to damage to the microvascularization of the retina as a result of prolonged exposure to metabolic changes induced by diabetes. The etiology of this complex disease remains unclear and poorly understood. It is associated with environmental and genetic factors. Almost all patients with Type 1 diabetes mellitus (T1DM) and more than 60% of T2DM patients are expected to have some type of retinopathy within the first 10 years after diagnosis of diabetes. Several genes have a possible role in DR. These genes are part of different physiological and pathophysiological processes in the body, often associated with inflammation, such as renin-angiotensin-aldosterone system, glucose-induced pathways, remodeling of the extracellular matrix, vascular endothelial dysfunction and angiogenesis (50). Vascular endothelial growth factor A (*VEGFA*) plays a role in angiogenesis and has been linked to DR by inducing hyperpermeability of retinal vessels, breakdown of the blood-retinal barrier, and neovascularization following microvascular changes that lead to hypoxia (38). In this study, the most significantly associated SNP with DR was rs3024997. However, in this study, rs3024997 was the only SNP associated with DR after adjustment for the effects of other associated markers with DR, indicating that rs3024997 is the key associated SNP in the Emirati population. This SNP was previously linked with macular degeneration (51), as well as coronary artery disease in a Chinese population (52). The association between DR and rs3024997 was not reported in previous studies, making this a novel finding for DR in our Emirati cohort. Two other SNPs in the *VEGFA* gene have been shown a borderline association with retinopathy, namely rs833068, and rs3024998. Furthermore, the SNP rs833068 was mentioned in a study of 554 T diabetic patients of Caucasian European descent, and highly associated with DR in T1DM patients (53) suggesting a possible common genetic association for retinopathy across the different types of diabetes. It has been shown that the expression of the VEGF protein is influenced by the SNPs of the *VEGFA* gene, in particular in the promoter region (54, 55) and the levels of VEGF protein are elevated in proliferative DR patients (56–58). The rs833068 in T1DM are in linkage disequilibrium with the promoter region of *VEGFA*, directly influencing protein expression and therefore vitreous concentrations of VEGF (38). Finally, the rs3024998 SNP was not reported in any previous studies, including T2DM or DR, making this another new finding.

### Diabetic Nephropathy

DNp is the leading cause of end-stage kidney disease (59), and the annual global death attributable to diabetes mellitus, including cardiovascular and renal complications, is nearly 3 million (60). A better understanding of the etiology and pathogenesis of DNp is needed to improve screening strategies, optimize therapies, and ultimately improve individual outcomes. *NOS3* influences plasma levels of nitric oxide metabolites and has been suggested to be involved in chronic renal failure (61). Oxidative stress is a major contributor to diabetic microvascular complications, and several groups have previously reported inconclusive data for the association between selected SNPs in the *NOS3* gene and diabetic nephropathy (62). In our study two SNPs rs743507 and rs1808593 related to the *NOS3* gene were found to have a borderline significant correlation to the development of DNp in the Emirati cohort investigated in this study. This is in partical agreement with a study by Mollsten et al. (63), where SNP rs743507 was associated with nephropathy in the Danish Caucasian cohort of Type 1 Diabetes patients but did not establish an association with neuropathy. An antisense mRNA induced by hypoxia (64) and transcribed from the opposite DNA strand of the NOS3 gene, overlapping with the 3’-end of the gene, has been suggested to participate in the post-transcriptional regulation of Endothelial-derived nitric oxide synthase (eNOS) encoded by the NOS3-gene (65). This type of regulation could be affected by SNPs at the 3’-end of the gene such as rs743507. Furthermore, to our knowledge, only one research has been published indicating no significant correlation of rs1808593 with DNp (41). The rs2070744 SNP in the upstream/promoter region of NOS3 has been shown to have functional activity; the C allele of rs2070744 is associated with reduced mRNA levels of NOS3 (66). It is interesting to note that a scientific group reported strong linkage disequilibrium between rs2070744 and rs891512 (67), and this variant shows r2 = 0.8 with rs1808593 which showed a significant association in our study.

However, in this study, we have identified a strong connection between the rs743507 of *NOS3* and the rs2020927, rs3905000 SNPs of the *ABCA1* gene when associated with DNp (p-value = 0.009). Additionally, rs3905000 was the only SNP associated with DNp after adjustment for the effects of other associated markers. This result suggest that rs743507 and rs3905000 are the key associated SNP with DNp in the Emirati population. SNP rs3905000 is evident in previous studies as an important genetic factor contributing to dyslipidemia and cardiovascular complications (68). Also, studies conducted in the United States have shown that the combination of hypertension and diabetic nephropathy is a leading cause of chronic kidney disease and, eventually, end-stage renal disease (69). Our results indicate, for the first time, the direct association between rs3905000 SNP and the progression of diabetic comorbidities.

### Diabetic Peripheral Neuropathy

DPN is a complex disorder emerging from the imbalance between multiple predisposing and protective genetic variants as well as from the interaction with environmental factors (70). Although, chronic hyperglycemia and duration of diabetes are the main contributors to the development of DPN, however, endothelial dysfunction has been shown to be an important pathophysiologic parameter for DPN (71). The modulation of the nitric oxide synthase (NOS) enzymes responsible for NO synthesis contribute to endothelial dysfunction (72, 73). Moreover, previous studies suggest that dysfunctional eNOS might play a critical role in the pathogenic pathway leading to diabetic microvascular complications including DPN (74, 75). As the *NOS3* gene is expressed in vascular smooth muscle, its ability to induce platelet aggregation can promote blood clotting, contributing to limiting blood supply to the peripheral circulation. Lack of blood supply in the peripheral microvasculature leads to nerve damage (76). Moreover, as *NOS3* contributes to systemic vasodilation to increase blood supply, a polymorphism such as associated with the current SNPs affect the function of the *NOS3* gene that can further worsen the peripheral neuropathy. Our findings showed that rs4496877 in the *NOS3* gene was strongly associated with DPN in the Emirati population. In addition, the rs4496877 was the only associated SNP with DPN after adjustment for the effects of other associated markers with DPN, making the rs3024997 a key associated SNP in the Emirati population. Furthermore, to our knowledge, no research has been published indicating a correlation of rs4496877 with neuropathy, marking this connection, a new finding that may be population specific.

### Diabetic Retinopathy/Nephropathy

This work presents the first study to investigate the genetic variations influencing the combinations of diabetic complications (DR, DNp, DPN) among T2DM patients of Arab origin from the United Arab Emirates. In the case of rs833061, previous work including a meta-analysis of 11 studies in an Asian cohort demonstrates a significant association of this polymorphism with increased DR susceptibility (35). Another study, including 500 Chinese T2DM patients with and without DR, also reported significant associations with this SNP in their patient group (77). However, a positive association with DNp was observed for the rs833061 SNP in the Northern Ireland population (78). Thus confirming our findings of borderline association between rs833061 SNP and dual diabetes complication DR/DNp.

All the previously mentioned SNPs are in the *VEGFA* gene, which is located in the short arm of chromosome 6 (6p21.3) and is highly polymorphic (24). *VEGFA* signals and regulates endothelial cell proliferation and migration of endothelial cells, which in turn leads to the formation of new vascular structures. This is why any upregulation in the expression of this gene could lead to microvascular pathologies such as retinopathy, nephropathy, and peripheral neuropathy, especially in T2DM patients (79, 80). For example, patients with retinopathy have under perfused retinas, potentially due to overall hypertension and dyslipidemia that lead to decreased perfusion of the retinal vessels. This would then lead to hypoxia, which immediately signals blood components to express *VEGFA* to induce angiogenesis and re-perfuse the retina (45, 46). Such a phenomenon is also evident in the rest of the diabetic comorbidities (nephropathy and peripheral neuropathy) as it is a systemic mechanism of salvaging the hypoxic tissue. This explains why our results show not only significant associations with SNPs in the *VEGFA* gene with retinopathy by itself, but also with the dual complications DR/DNp for rs3024997 and rs833068 of the *VEGFA* gene. Additionally, we have identified a strong connection between the rs3024997 and rs833068 of the *VEGFA* gene and the rs4496877 SNP of the *NOS3* gene when associated with DR/DNp. This result suggest that the rs4496877 marker may has a modifying effect on the risk of DR/DNp instigated by the markers rs3024997 and rs833068. In the literature, only one previous study showing no significant association between rs3024997 of the *VEGFA* gene and DNp in the European population (39). On the other hand, the *VEGFA* SNP rs833068 was associated with DNp in the Korean population (37).

### Diabetic Peripheral Neuropathy/Nephropathy and Retinopathy/Peripheral Neuropathy/Nephropathy

Two SNPs rs4149263 and rs2230806 in the *ABCA1* gene were found to have a borderline significant association with DPN/DNp and DR/DPN/DNp combinations in the studied cohort, respectively. However, in this study, rs4149263 was the only SNP associated with DPN/DNp after adjustment for the effects of other associated markers, indicating that rs4149263 is the key associated SNP in the Emirati population. Additionally, we have identified a strong connection between the rs2230806 and the other two SNPs of the *ABCA1* gene rs4149263, rs3905000 when associated with DR/DPN/DNp. This result suggest that the markers rs4149263, rs3905000 may have a modifying effect on the risk of DR/DPN/DNp instigated by the marker rs2230806. As for rs4149263, previous study has established an association between this respective SNP and dyslipidemia (81). Furthermore, the SNP rs2230806 in the *ABCA1* gene were found to be not correlated with T2DM in Southeast of Iran. According to our research, no previous studies have associated rs4149263 to DPN/DNp and rs2230806 to DR/DPN/DNp, making this a novel finding for diabetic complications in our Emirati cohort.

The rising levels of cholesterol in the case of *ABCA1* mutations leading to various SNPs and thus, variable protein product expression will inevitably lead to dyslipidemia, renal insufficiency, and diabetic nephropathy. The metabolic disturbances resulting from nephropathy will lead to hemodynamic fluctuation, extensive Renin-Angiotensin-Aldosterone System (RAAS) activation, and subsequently to hypertension as the body attempts to raise GFR (69, 81, 82). A polymorphic *ABCA1* is thus associated with albuminuria, dyslipidemia, and hypertensive crises. The elevation in blood pressure can then lead to complications in peripheral vessels, explaining why the *ABCA1* polymorphisms studied are associated with all three diabetic comorbidities (69, 82, 83). Hypertension and dyslipidemia can lead to occlusions of peripheral vessels, leading to the progression of neuropathy in the presence of nephropathy. The association between these SNPs and diabetic complications can be due to the excess oxidative stress brought forth by hyperglycemia, which causes vascular dysfunction and leads to retinal neovascularization in combination with *VEGF* activity. *VEGF* induced angiogenesis combined with dyslipidemia due to *ABCA1* polymorphisms will inevitably lead to retinopathy. The same effect can be observed in the peripheral blood vessels, excessive cholesterol deposits due to *ABCA1* dysfunction, can cause peripheral neuropathy and angiogenesis (68, 83). This then explains why results from the current study showed a gene-gene interaction between *ABCA1*, *NOS3* when associated with DN and *VEGFA*, *NOS3* when associated with DR/DNp and why GeneMANIA software is showing direct protein-protein interaction between VEGFA and NOS3 and indirect gene-gene interaction between *ABCA1* and NOS3 genes. Further studies are needed to elucidate the functions of the polymorphisms involved in the different groups of diabetes complications.

### Comparaison of Allele Frequencies With Other Ethnic Groups

The comparison of our data in this study with European populations showed differences at 5 among the 9 top significant SNPs that were compared. In addition, these results report differences in genetic data between the Emirati, African East Asian and American populations. These findings are consistent with a previous study that showed the estimated ethnic background of two whole Emirati genomes to be differ from European, African and American groups (84). The difference between these populations could be due to the geographical distance between the lands on which they reside.

## Summary and Future Work

In conclusion, our results corroborate previous findings and add new results. We report associations of four SNPs in *VEGFA* and two SNPs in *NOS3* with DR, DNp and DR/DNp attributed to their role in angiogenesis and oxidative stress. Moreover, associations between DNp and DN/DNp and *ABCA1* and *NOS3* SNPs were common due to their influence on dyslipidemia and hypertension, which ultimately reduces kidney function. Finally, some links between DN and DR/DNp were found with *NOS3*, and *VEGFA*, mainly due to the number of factors and genes interactions that complement each other leading to diabetes disease progression.

A limitation of this study would be the small sample size (n = 158) and that the logistic analyses performed did not include treatment modalities which may have affected the analyses due to multiple drug class adjustments. Furthermore, in view of the population-specific genomic variations and the high prevalence of T2DM in the Emirati population, larger and more widely replication study is important to validate the current findings in order to achieve highly specific results and allow proper genetically guided early detection and personalized medicine for T2DM patients in the future.

## Data Availability Statement

The original contributions presented in the study are included in the article/**Supplementary Material**. Further inquiries can be directed to the corresponding author.

## Ethics Statement

The studies involving human participants were reviewed and approved by Institutional Ethics Committee of the hospital (SKMC-IRB-Ref R292). The patients/participants provided their written informed consent to participate in this study.

## Author Contributions

HSA, HJ, WA and AK: Conceptualization, project administration, data analysis and interpretation, writing of original draft. SC, HJ, NS and HA: Data analysis, conceptualization, writing and editing. All authors have read and agreed to the published version of the manuscript.

## Funding

The project was funded by internal funds provided by Khalifa University awarded to Alsafar H KUIRFL2 in 2015.

## Conflict of Interest

The authors declare that the research was conducted in the absence of any commercial or financial relationships that could be construed as a potential conflict of interest.

## Publisher’s Note

All claims expressed in this article are solely those of the authors and do not necessarily represent those of their affiliated organizations, or those of the publisher, the editors and the reviewers. Any product that may be evaluated in this article, or claim that may be made by its manufacturer, is not guaranteed or endorsed by the publisher.
