# Supplementary material for: Genetic Variants and Their Associations to Type 2 Diabetes Mellitus Complications in the United Arab Emirates
Source: Front Endocrinol (Lausanne). 2022 Jan 6;12:751885. doi: 10.3389/fendo.2021.751885 (PMC8772337; doi:10.3389/fendo.2021.751885)
Supplement: Supplementary file 1 [file Table_1.docx]

**Supplementary table 1:** Genotype, allele frequencies and Hardy-Weinberg equilibrium tests for SNPs based on the study subjects.

| **Genes** | **SNPs** | **Genotypes** | **Frequency (%)** | **Minor/Major Allele** | **MAF (%)** | ***p-*HWE T2DM without complications** | ***p-*HWE**  **T2DM with complications** |
| --- | --- | --- | --- | --- | --- | --- | --- |
| ***SLC2A1*** | rs841853 | GG | 53.50 | T/G | 29.30 | 0.572 |  |
|  |  | GT | 34.39 |  |  |  | 0.041 |
|  |  | TT | 12.10 |  |  |  |  |
| ***VEGFA*** | rs833061 | TT | 14.72 | C/T | 60.9 | 0.594 | 0.458 |
|  |  | TC | 48.72 |  |  |  |  |
|  |  | CC | 36.54 |  |  |  |  |
|  | rs833068 | GG | 37.18 | A/G | 40.06 | 0.077 | 0.863 |
|  |  | GA | 45.51 |  |  |  |  |
|  |  | AA | 17.31 |  |  |  |  |
|  | rs3024997 | GG | 40.76 | A/G | 37.58 | 0.145 | 0.843 |
|  |  | GA | 43.31 |  |  |  |  |
|  |  | AA | 15.92 |  |  |  |  |
|  | rs3024998 | CC | 43.31 | T/C | 35.99 | 0.246 | 0.466 |
|  |  | CT | 41.40 |  |  |  |  |
|  |  | TT | 15.29 |  |  |  |  |
| ***NOS3*** | rs4496877 | GG | 7.69 | T/G | 73.40 | 0.380 | 0.992 |
|  |  | GT | 37.82 |  |  |  |  |
|  |  | TT | 54.49 |  |  |  |  |
|  | rs743507 | AA | 5.16 | G/A | 78.40 | 0.181 | 0.322 |
|  |  | AG | 32.90 |  |  |  |  |
|  |  | GG | 61.94 |  |  |  |  |
|  | rs1808593 | TT | 5.10 | G/T | 78.66 | 0.181 |  |
|  |  | TG | 32.48 |  |  |  | 0.297 |
|  |  | GG | 62.42 |  |  |  |  |
| ***ABCA1*** | rs4149339 | CC | 52.87 | T/C | 27.39 | 0.230 |  |
|  |  | CT | 39.49 |  |  |  | 0.720 |
|  |  | TT | 7.64 |  |  |  |  |
|  | rs2020927 | TT | 43.31 | C/T | 33.44 | 0.572 | 0.342 |
|  |  | TC | 46.50 |  |  |  |  |
|  |  | CC | 10.19 |  |  |  |  |
|  | rs2230806 | GG | 30.57 | A/G | 44.59 | 0.625 | 0.914 |
|  |  | GA | 49.68 |  |  |  |  |
|  |  | AA | 19.75 |  |  |  |  |
|  | rs2249891 | AA | 56.05 | G/A | 25.80 | 0.230 | 0.312 |
|  |  | AG | 36.31 |  |  |  |  |
|  |  | GG | 7.64 |  |  |  |  |
|  | rs4149268 | AA | 26.11 | G/A | 48.09 | 0.875 | 0.560 |
|  |  | AG | 51.59 |  |  |  |  |
|  |  | GG | 22.29 |  |  |  |  |
|  | rs3905000 | GG | 65.61 | A/G | 18.15 | 0.344 | 0.364 |
|  |  | GA | 32.48 |  |  |  |  |
|  |  | AA | 1.91 |  |  |  |  |
|  | rs4149263 | TT | 54.14 | C/T | 26.75 | 0.344 | 0.720 |
|  |  | TC | 38.22 |  |  |  |  |
|  |  | CC | 7.64 |  |  |  |  |
| ***TGFB1*** | rs1800469 | CC | 42.04 | T/C | 35.99 | 0.686 | 0.699 |
|  |  | CT | 43.95 |  |  |  |  |
|  |  | TT | 14.01 |  |  |  |  |
|  | rs4803457 | CC | 31.85 | T/C | 42.99 | 0.572 | 0.914 |
|  |  | CT | 50.32 |  |  |  |  |
|  |  | TT | 17.83 |  |  |  |  |
| ***COMT*** | rs933271 | TT | 51.59 | C/A | 29.62 | 0.013 | 0.798 |
|  |  | TC | 37.58 |  |  |  |  |
|  |  | CC | 10.83 |  |  |  |  |

Values in *bold* indicate significant *p*-value < 0.01 for HWE.

Abbreviations: SNP: single nucleotide polymorphism, MAF: minor allele frequency, HWE: Hardy-Weinberg equilibrium, *SLC2A1*: Solute Carrier Family 2 Member 1, *VEGFA*: Vascular Endothelial Growth Factor A, *NOS3*: Nitric Oxide Synthase 3, *ABCA1*: ATP Binding Cassette Subfamily A Member 1, *TGFB1*: Transforming Growth Factor Beta 1, *COMT*: Catechol-O-Methyltransferase.
